# Phenotype-First Diagnostic Framework for Tracking Fluoroquinolone Resistance in *Escherichia coli*

**DOI:** 10.3390/diagnostics15222831

**Published:** 2025-11-07

**Authors:** Eman Marzouk, Abdulaziz M. Almuzaini

**Affiliations:** 1 Department of Public Health, College of Applied Medical Sciences, Qassim University, P.O. Box 6666, Buraydah 51452, Saudi Arabia; 2 Department of Veterinary Preventive Medicine, College of Veterinary Medicine, Qassim University, Buraydah 51452, Saudi Arabia

**Keywords:** fluoroquinolone resistance, *Escherichia coli*, antibiotic resistance, collateral resistance, phenotype-first approach, antimicrobial stewardship

## Abstract

**Background:** Fluoroquinolone (FQ) resistance in *Escherichia coli* (*E. coli*) undermines empiric therapy and often coincides with multidrug resistance (MDR). Because sequencing is not routinely available in many laboratories, we evaluated a phenotype-first, sequencing-independent diagnostic framework deployable on standard platforms. **Methods:** We profiled 45 archived *E. coli* isolates for susceptibility (Clinical and Laboratory Standards Institute [CLSI]-guided), extended-spectrum β-lactamase (ESBL) and AmpC β-lactamase (AmpC) phenotypes, MDR, and multiple-antibiotic resistance (MAR) indices. Ten founders (five FQ-susceptible [FQ-S], five low-level resistant [LLR]) seeded 20 parallel lineages exposed to stepwise ciprofloxacin. We tracked minimum inhibitory concentrations (MICs), collateral resistance, growth kinetics, and biofilm biomass using matrix-assisted laser desorption/ionization time-of-flight mass spectrometry (MALDI-TOF MS) for identification, automated and reference antimicrobial susceptibility testing (AST), growth-curve analysis, and crystal violet microtiter assays. The intended use is a sequencing-independent workflow for routine laboratories—especially where whole-genome sequencing is not readily available—working with archived or prospective clinical *E. coli*. This workflow is best applied when local FQ nonsusceptibility threatens empiric reliability; inputs include standard ID/AST with simple growth and biofilm assays. Primary outputs include: (i) MIC trajectories with time to high-level resistance (HLR), (ii) ΔMAR-summarized collateral resistance with class-level susceptible-to-resistant conversions, and (iii) concise fitness/biofilm summaries to guide empiric-policy refresh and early de-escalation. **Results:** At baseline, ciprofloxacin nonsusceptibility was 40.0%; ESBL and AmpC phenotypes were confirmed in 28.9% and 15.6%, respectively; 46.7% met the MDR definition; and the median MAR index was 0.29. During evolution, 70% of lineages reached HLR (MIC ≥ 4 μg/mL), with earlier conversion from LLR versus FQ-S founders (median 7 vs. 11 passages). Collateral resistance emerged most often to third-generation cephalosporins (3GCs), trimethoprim–sulfamethoxazole, and tetracyclines, while carbapenem activity was preserved. MAR increased in parallel with rising MICs. Resistance acquisition imposed modest fitness costs (slightly reduced growth rates and longer lag phases) that were partly offset under subinhibitory ciprofloxacin, whereas biofilm biomass changed little. **Conclusions:** this phenotype-first, routine-laboratory workflow rapidly maps FQ resistance and clinically relevant co-selection in *E. coli*. In high-resistance settings, empiric FQ use is difficult to justify, and MAR trends provide practical co-selection signals for stewardship. This reproducible framework complements genomic surveillance and is directly applicable where sequencing is unavailable.

## 1. Introduction

Fluoroquinolones (FQs) have long been mainstays for the management of *Escherichia coli* (*E. coli*) infections because of their excellent oral bioavailability, deep tissue penetration, and dependable Gram-negative activity—particularly in urinary tract, intra-abdominal, and bloodstream infections [1]. Sustained selection pressure across human, veterinary, and environmental domains has driven a global rise in FQ resistance, undermining empiric therapy and complicating infection control [2]; ecological analyses further link community FQ consumption with accelerated *E. coli* resistance [3]. The World Health Organization (WHO) Bacterial Priority Pathogens List (BPPL 2024) highlights the public-health threat posed by resistant *E. coli* and related Enterobacterales, while Global Antimicrobial Resistance and Use Surveillance System (GLASS) analyses document substantial national burdens and upward resistance trajectories with clear implications for stewardship and outcomes [4].

Across regions, surveillance and systematic reviews consistently report double-digit FQ nonsusceptibility in *E. coli* causing community- and healthcare-associated urinary tract infections (UTIs), with marked heterogeneity by setting. In many cohorts, ciprofloxacin resistance in community-acquired uncomplicated UTI exceeds the 10% threshold—often substantially—eroding the reliability of FQs for empiric therapy [5]. These trends frequently coincide with resistance to third-generation cephalosporins (3GCs) and other oral agents, a pattern mirrored in multi-country surveillance showing persistently high *E. coli* burdens despite wide interregional variability [6]. A recent systematic review focused on community-acquired UTI corroborates this picture, highlighting widespread FQ resistance and its convergence with broader multidrug-resistant phenotypes, thereby narrowing effective oral options and complicating stewardship and infection-control decisions [7].

Mechanistically, FQ resistance in *E. coli* typically arises through stepwise mutations in the quinolone resistance–determining regions of DNA gyrase (gyrA, gyrB) and topoisomerase IV (parC, parE), coupled with reduced intracellular drug accumulation via multidrug efflux (notably AcrAB–TolC) and decreased permeability; in parallel, plasmid-mediated determinants—qnrA/qnrB/qnrS, aac(6′)-Ib-cr, qepA, and oqxAB—can elevate minimum inhibitory concentrations (MICs) and foster subsequent selection of high-level chromosomal resistance under FQ exposure [8,9]. We reference these mechanisms as background; importantly, our study is phenotype-focused and did not perform targeted or whole-genome sequencing (WGS) to assign molecular mechanisms directly.

Resistance emergence and maintenance are further shaped by bacterial fitness and ecological context: many resistance mutations impose measurable fitness costs—slower growth, prolonged lag, and reduced competitiveness—that may be offset by compensatory evolution, allowing resistant lineages to persist even when antibiotic pressure wanes [10]. In *E. coli*, the resistance–nodulation–division (RND) efflux system AcrAB–TolC (encoded by acrA, acrB, and tolC) is a major driver of multidrug phenotypes and persistence; pharmacologic efflux inhibition with phenyl-arginine-β-naphthylamide (PAβN) reduces FQ tolerance in stationary-phase cells, underscoring the central contribution of efflux to the FQ-resistant state [11].

Biofilm biology adds another layer: continuous FQ exposure within biofilms accelerates resistance evolution, increases MICs, and heightens recalcitrance to therapy [12]. The microtiter crystal-violet assay remains a robust, widely adopted method for quantifying biofilm biomass and comparing phenotypes across strains or conditions [13]. From an epidemiologic perspective, the global success of high-risk extraintestinal pathogenic *E. coli* (ExPEC) clones—most notably ST131 (clade C/H30R) and the more recently emergent ST1193—has been tightly linked to FQ resistance and spread in both healthcare and community settings [14], with evidence of cross-compartment transmission and near-identical resistance/virulence plasmids bridging animal and human sources [15].

Despite extensive genomics work on FQ-resistant *E. coli*—spanning mutational spectra, mobile elements, and clonal dynamics—many laboratories lack routine access to whole-genome sequencing (WGS) because of costs, infrastructure, and standardization hurdles. There is therefore a practical need for phenotype-first frameworks that track resistance emergence over time with accessible methods and quantify collateral phenotypes such as co-resistance, fitness costs, and biofilm changes [16,17]. Recent studies that chart FQ resistance trajectories commonly incorporate genomic confirmation—for example, delineating SOS-response–dependent pathways—underscoring the value of complementary, routine laboratory approaches deployable without [18].

Widely available clinical microbiology tools can support high-quality, standardized studies of this phenotype-first question. Matrix-assisted laser desorption/ionization time-of-flight mass spectrometry (MALDI-TOF MS) provides rapid, accurate species-level identification and has reshaped routine workflows; pairing conventional morphology/biochemical identification with MALDI confirms *E. coli* robustly across resource-diverse settings [19,20,21,22]. Vitek 2 offers automated antimicrobial susceptibility testing (AST) with performance comparable to reference broth microdilution for Enterobacterales [23]. Meanwhile, the Clinical and Laboratory Standards Institute (CLSI) M100 provides harmonized breakpoints and quality-control parameters that standardize interpretation across laboratories [24]. Together with classical methods—CLSI-guided disk diffusion and broth microdilution, routine growth-curve analysis, and the microtiter crystal-violet biofilm assay—these platforms enable standardized, reproducible measurement of phenotypic resistance trajectories and collateral traits in routine laboratories [25,26].

Study aim and approach. We present a sequencing-independent, routine-laboratory framework for settings working with archived clinical *E. coli* isolates (as in this study) and readily extensible to prospective collections. Apply it when local FQ nonsusceptibility is a concern (e.g., nearing or exceeding empiric thresholds) and stewardship needs lab-anchored evidence on how quickly resistance amplifies under ciprofloxacin pressure. Eligible founders include both FQ-susceptible (FQ-S) and low-level-resistant (LLR) isolates. Using MALDI-TOF MS and CLSI-interpreted AST—augmented by simple growth-curve and crystal-violet biofilm assays—the framework delivers four actionable outputs: (i) FQ MIC trajectories with a prespecified time-to-high-level resistance (HLR) endpoint; (ii) collateral co-resistance summarized by ΔMAR with class-level susceptible-to-resistant conversions (notably 3GCs, trimethoprim–sulfamethoxazole [TMP–SMX], tetracyclines); (iii) concise fitness readouts (growth rate and lag); and (iv) biofilm biomass shifts. These decision-oriented readouts translate familiar phenotypic measurements into stewardship signals that support empiric-policy refresh, early de-escalation/rotation, and early identification of lineages at higher risk of rapid conversion to HLR—without requiring sequencing.

In short, we offer a sequencing-free, phenotype-first framework that tracks resistance amplification over time and captures collateral resistance bundles using only routine clinical tools. The approach yields actionable stewardship indicators—notably that MAR rises in parallel with ciprofloxacin MICs and that LLR founders convert to HLR faster—which are directly useful for empiric-therapy policies and de-escalation, making the workflow transferable to diverse routine laboratories and a practical complement to genomic surveillance.

## 2. Materials and Methods

### 2.1. Study Design and Oversight

We conducted a two-arm, laboratory-based study. Arm 1 profiled 45 archived *E. coli* isolates using routine species identification and AST to define FQ resistance, co-resistance (including ESBL phenotypes), and MAR indices. Arm 2 applied in vitro experimental evolution, passaging selected founders through stepwise ciprofloxacin to measure MIC trajectories, growth fitness, biofilm phenotypes, and post-withdrawal stability. Arm 1 ran from December 2024 to February 2025; Arm 2 ran from March 2025 to July 2025. All work was performed in a biosafety level 2 (BSL-2) laboratory under institutional procedures. Because only pre-existing, de-identified isolates and in vitro experiments were used, Institutional Review Board (IRB) and Institutional Biosafety Committee (IBC) approvals were not required under local policy. Quality assurance followed manufacturer instructions and CLSI M100 standards; *E. coli* ATCC 25922 served as the batch quality control (QC) strain. Replicate lineages, prespecified thresholds/endpoints, and standardized data templates were used, with blinded analysis for growth-curve and biofilm assays when applicable. Isolates were fully de-identified prior to research use; no patient identifiers or clinical metadata were accessed or retained.

Intended use, inputs, and outputs (routine-lab implementation). Designed for routine clinical microbiology laboratories—especially where WGS is not readily available—this workflow can be applied to archived or prospective clinical *E. coli*. Apply when local FQ nonsusceptibility is a concern and stewardship requires trajectory-aware evidence. Using MALDI-TOF MS identification and CLSI-interpreted AST, augmented by simple growth-curve and crystal-violet biofilm assays, the framework yields four decision-oriented outputs: (i) ciprofloxacin MIC trajectories with a prespecified time-to-HLR endpoint; (ii) collateral co-resistance summarized by ΔMAR with class-level S → R conversions (notably 3GCs, TMP–SMX, tetracyclines); (iii) concise fitness readouts (growth rate and lag); and (iv) biofilm biomass shifts—informing empiric-policy refresh, early de-escalation/rotation, and early identification of lineages at higher risk of rapid conversion to HLR, without sequencing.

### 2.2. Isolates and Inclusion Criteria

We retrieved 45 archived *E. coli* isolates from the departmental biobank, stored in commercial cryobank bead vials at −80 °C. For each isolate, a single bead was aseptically transferred to prepare a working culture; the parent vial remained at −80 °C to avoid freeze–thaw cycling. Revival and purity were confirmed on blood agar and MacConkey agar, with verification of uniform colony morphology and a Gram-negative rod smear. All eligibility checks—viability, unequivocal species identification, non-duplication, and biomass sufficiency—were completed before any experimental manipulation to ensure downstream outputs reflect routine-lab feasibility rather than strain-selection bias.

Inclusion criteria: (i) viability on subculture within two attempts; (ii) unequivocal species identification as *E. coli* by MALDI-TOF MS, supplemented by conventional phenotypic tests when needed (Section 2.3); and (iii) sufficient biomass to complete AST and planned phenotypic assays. Exclusion criteria: mixed cultures/contamination, ambiguous species identification, insufficient growth, or duplicate isolates from the same patient/episode when detectable from archive metadata. Non-duplicate status was enforced by archive metadata, and isolates were treated as unique patient/episode entries. To minimize laboratory adaptation, isolates underwent no more than two passages before baseline testing, and a single well-isolated colony initiated each assay. The resulting 45 non-duplicate, viable, unequivocally identified *E. coli* comprised the Arm 1 cohort and the eligibility pool for founder selection in the experimental-evolution arm (Section 2.6).

### 2.3. Species Identification (Conventional Methods Followed by MALDI-TOF MS)

Primary identification used conventional methods: colony morphology on MacConkey agar (lactose-fermenting pink/red colonies typical of *E. coli*) and, when indicated, eosin methylene blue (EMB) agar (dark colonies with a metallic-green sheen); Gram stain (Gram-negative rods); and the indole–methyl red–Voges–Proskauer–citrate (IMViC) profile with triple sugar iron (TSI) and oxidase/catalase tests, interpreted per standard protocols (all media/reagents: Sigma-Aldrich, St. Louis, MO, USA) [27]. Species-level confirmation was then obtained using MALDI-TOF MS (Microflex LT, Bruker Daltonics; Compass FlexSeries v1.3, Biotyper workflow) by the direct-smear method with HCCA matrix; daily calibration employed the Bruker Bacterial Test Standard (BTS; *E. coli*). A log(score) ≥ 2.0 with concordant duplicate spots was required for species identification; lower or discordant scores prompted repeat spotting (with on-plate formic-acid extraction as needed) and/or repeat conventional testing until a single, unequivocal result was obtained. This workflow and score threshold align with widely adopted Bruker Biotyper practices and published evaluations of MALDI-TOF MS performance for rapid species-level identification in clinical laboratories [20]. To ensure routine-lab transferability while keeping the workflow sequencing-independent, we mandated a log(score) ≥ 2.0 in duplicate before advancing any isolate.

### 2.4. Antimicrobial Susceptibility Testing (AST)

AST was performed on the Vitek 2 Compact (bioMérieux, Marcy-l’Étoile, France) using the Gram-negative AST-N439 card for Enterobacterales. Inocula were adjusted to 0.5 McFarland in sterile saline and processed per the manufacturer’s instructions, with results reviewed in the Advanced Expert System (AES). The AST-N439 panel (verified from the local package insert) included the primary FQs—ciprofloxacin and levofloxacin—and key comparators for *E. coli* (ampicillin; amoxicillin–clavulanate; third-/fourth-generation cephalosporins cefotaxime, ceftazidime, ceftriaxone, cefepime; piperacillin–tazobactam; imipenem; meropenem; gentamicin; amikacin; TMP–SMX; tetracycline-class agents; and nitrofurantoin). Operational definitions: HLR was prespecified as ciprofloxacin MIC ≥ 4 µg/mL or two consecutive “Resistant” calls on automated AST, enabling a time-to-event endpoint (time-to-HLR). In parallel, ΔMAR and class-level S → R conversions (3GCs, TMP–SMX, tetracyclines; carbapenems tracked as an internal specificity check) were computed to summarize collateral co-selection in a stewardship-ready format.

Interpretive categories (S/I/R) followed CLSI M100 breakpoints current at the time of testing, and *E. coli* ATCC 25922 was included in each run for QC [28]. For FQ confirmation, disk diffusion (ciprofloxacin, levofloxacin) was performed on Mueller–Hinton agar (Sigma-Aldrich, USA) and read at 16–18 h; discrepancies between Vitek 2 and disk diffusion were resolved by broth microdilution and, where appropriate, Etest MICs, with the reference method reported. The choice of AST-N439 is supported by independent evaluations demonstrating Vitek 2 GN card performance comparable to reference methods for Enterobacterales, including specific assessments of this card [29]. All AST interpretations followed CLSI M100; when methods disagreed, the CLSI reference broth microdilution result was reported.

### 2.5. ESBL/AmpC Phenotypes, Multidrug Resistance (MDR), and MAR Index

Putative ESBL producers flagged by the Vitek 2 AES were confirmed using the CLSI phenotypic confirmatory disk test with ceftazidime (30 µg) and cefotaxime (30 µg) with and without clavulanate, read at 16–18 h; a ≥5 mm increase in zone diameter with clavulanate for either agent was interpreted as ESBL-positive [28]. AmpC screening used cefoxitin (30 µg) nonsusceptibility as a surrogate marker; screen-positive isolates underwent inhibitor-based phenotypic evaluation (e.g., boronic-acid augmentation of cephalosporins) and/or cefoxitin–cloxacillin double-disk synergy (CC-DDS) to distinguish AmpC from ESBL activity [30]. MDR was defined as nonsusceptibility to at least one agent in ≥3 antimicrobial categories [31]. The MAR index was calculated as (number of antibiotics with a “Resistant” call) ÷ (total number tested); higher values were interpreted against historical source-risk benchmarks [32]. All interpretations followed the CLSI M100 edition current at the time of testing [28]. These operational definitions (ESBL confirmation, AmpC screening/confirmation, MDR, and MAR) were prespecified and are used verbatim throughout the Results and Tables.

### 2.6. Selection of Founders for Experimental Evolution

From the 45-isolate cohort, we selected 8–12 founder *E. coli* isolates to encompass a deliberate range of baseline phenotypes while preserving feasibility for parallel evolution. We selected ten founders (five FQ-susceptible [FQ-S], five low-level-resistant [LLR] per CLSI M100), stratifying by ciprofloxacin/levofloxacin susceptibility and balancing ESBL status (ESBL-positive and ESBL-negative). Two independent lineages were initiated per founder (20 lineages total). Additional inclusion criteria were unequivocal species-level identification by MALDI-TOF MS, stable growth on subculture with uniform colony morphology, sufficient biomass for repeated assays, and non-redundant antibiograms/MAR indices to avoid over-representation of similar profiles. To preserve evolutionary “headroom,” high-level FQ-resistant isolates were not selected as founders. Before initiating passages, we reconfirmed baseline FQ susceptibility using broth microdilution MICs, Vitek 2 results, and disk-diffusion zone diameters, all from a single well-isolated colony after ≤2 laboratory passages. Cryopreserved baseline aliquots (−80 °C, bead system) were prepared for each founder to anchor downstream comparisons. Founder selection was intentionally grounded in routine-lab realism—FQ-S and LLR sources, balanced ESBL status, and diverse but non-redundant antibiograms—to ensure that subsequent readouts (time-to-HLR trajectories, ΔMAR-summarized collateral bundles, and concise fitness/biofilm shifts) reflect decision needs in everyday clinical practice rather than genomically pre-curated strain behavior.

### 2.7. Experimental Evolution Under Ciprofloxacin

Baseline ciprofloxacin MICs were determined by broth microdilution in cation-adjusted Mueller–Hinton broth (CA-MHB) and, where available, cross-checked on Vitek 2. For each founder, a single well-isolated colony was used to start an overnight culture; mid-log inocula were standardized to approximately 1 × 10^6^ CFU/mL (OD_600_ ≈ 0.02–0.05) in CA-MHB. Two independent lineages were propagated per founder through a predefined stepwise ciprofloxacin series expressed as fold-increments relative to the founder MIC (e.g., 0.25-fold, 0.5-fold, 1-fold, 2-fold, 4-fold, 8-fold) with absolute concentrations specified in µg/mL for reproducibility (e.g., for susceptible founders with MIC 0.06–0.125 µg/mL: ~0.03 → 0.06 → 0.125 → 0.25 → 0.5 → 1.0 µg/mL; then 2, 4, and 8 µg/mL if growth persisted). Cultures (5 mL) were incubated at 37 °C with orbital shaking (~180 rpm) for 18–24 h per step.

This fold-relative schedule mimicked clinically relevant escalation while keeping results portable across routine labs. Primary decision outputs were prespecified as (i) time-to-HLR (passages-to-event), (ii) ΔMAR with class-level S → R conversions (3GCs, TMP–SMX, tetracyclines; carbapenems tracked as a specificity check), and (iii) simple fitness/biofilm shifts—such that archived (as here) or prospective clinical collections yield immediately interpretable stewardship signals without sequencing. Progression required visible growth in ≥2 technical replicates and absence of contamination on purity streaks; failure prompted one retreat passage before re-challenge, with persistent failure recorded as lineage extinction. After each successful passage and at endpoints, 1 mL aliquots were cryopreserved at −80 °C (CRYOBANK™ bead vials or 15–20% glycerol). Antibiotic-free control lineages were passaged in parallel. Evolution proceeded until a prespecified HLR threshold was reached—ciprofloxacin MIC ≥ 4 µg/mL or two consecutive “Resistant” calls on automated AST—or until 30 daily passages, or lineage extinction.

### 2.8. Post-Selection Stability and Compensatory Adaptation

Endpoint clones were passaged daily for 10 transfers in antibiotic-free CA-MHB at 37 °C with orbital shaking (~180 rpm), starting from a single purified colony (≤2 passages). Aliquots were archived at T0 (immediately post-evolution) and T10 (after withdrawal). After T10, we repeated AST (Vitek 2), disk diffusion (ciprofloxacin, levofloxacin), and broth microdilution MICs to assess resistance stability or reversion; stability was defined a priori as an MIC change of ≤1 two-fold dilution with unchanged CLSI S/I/R category [28]. We then re-evaluated growth kinetics (μmax, lag time, area under the curve [AUC]) and biofilm biomass (crystal-violet assay) to detect compensatory adaptation versus true reversion [10]. *E. coli* ATCC 25922 was included as QC; endpoint and post-withdrawal aliquots were cryopreserved at −80 °C for traceability.

### 2.9. Growth Kinetics (Fitness) Assays

Overnight cultures were diluted in CA-MHB to a starting optical density of OD_600_ ≈ 0.02–0.05, then dispensed into clear, flat-bottom 96-well microplates (technical triplicates per condition; duplicate biological experiments on separate days). To minimize edge effects, outer wells were filled with sterile medium and sample positions randomized; plates were covered with breathable sealing film. Plates were incubated at 37 °C in a microplate reader with orbital shaking between reads, and OD_600_ was recorded every 10–15 min for 18–24 h. Medium-only wells served as blanks for background subtraction. From background-corrected curves, we derived μmax, lag time, and AUC using either peak slopes of ln(OD_600_) or nonlinear regression (modified Gompertz/logistic) in R or GraphPad Prism. Runs were accepted only if *E. coli* ATCC 25922 controls met prespecified ranges. Analyses were performed with blinded sample identifiers; software versions were R 4.5.1 and GraphPad Prism 10.6.1. The 24 h time point was designated the primary analysis window. These concise fitness readouts were prespecified to provide decision-facing context for resistance amplification—capturing potential trade-offs without specialized instrumentation—to ensure that results remain directly transferable to routine labs.

### 2.10. Biofilm Quantification

Biofilm biomass was quantified using the crystal-violet microtiter assay in clear, flat-bottom 96-well plates (technical triplicates; biological duplicates). Cultures were diluted to OD_600_ ≈ 0.02–0.05, dispensed (200 µL/well), and incubated statically at 37 °C for 24 h (and 48 h where indicated). The 24 h measurement was the prespecified primary endpoint. Wells were washed three times with phosphate-buffered saline (PBS), air-dried, stained with 0.1% crystal violet for 15 min, washed, and the bound dye was solubilized with 95% ethanol (or 80:20 ethanol/acetone) before reading A_570_. Biofilm signals were normalized to paired planktonic OD_600_ (Biofilm Index = A_570_/OD_600_) to account for growth differences [26]. We selected the Biofilm Index as a routine-friendly summary metric that complements MIC and ΔMAR—offering a structural phenotype most clinical laboratories can reproduce alongside standard ID/AST.

### 2.11. Outcomes and Definitions

We use the following terms throughout: ESBL; AmpC; MDR (nonsusceptible to ≥1 agent in ≥3 classes); MAR index; FQ-S founders; LLR founders; and HLR (ciprofloxacin MIC ≥ 4 µg/mL or two consecutive “Resistant” calls). Primary outcomes were (i) ciprofloxacin MIC trajectories (µg/mL and fold-change vs. baseline) and (ii) the proportion and passages to event of lineages achieving HLR, defined a priori as MIC ≥ 4 µg/mL or two consecutive “Resistant” calls on automated AST. These endpoints operationalize the intended use: time-to-HLR quantifies the speed of resistance amplification, while ΔMAR and class-level S → R conversions summarize collateral co-selection; paired fitness and biofilm metrics capture trade-offs—together yielding the actionable suite needed for empiric-policy refresh and early de-escalation in routine settings. Secondary outcomes included co-resistance acquisition (class- and agent-level), change in MAR index (endpoint and post-withdrawal vs. baseline), growth fitness (μmax, lag time, AUC), biofilm biomass (A_570_ and Biofilm Index), and post-selection stability after antibiotic withdrawal, classified as stable, reversion, or amplification. Extinct lineages were censored at the step of failure to regrow after retreat. All outcome definitions were finalized before data analysis and applied uniformly across lineages.

### 2.12. Data Handling and Statistical Analysis

Data were recorded contemporaneously in locked spreadsheets keyed by founder, lineage, and passage. Analyses were performed in IBM SPSS Statistics, GraphPad Prism, and R. Reporting convention: continuous outcomes are summarized as median (interquartile range, IQR) unless stated otherwise (means ± SD where appropriate); IQR denotes the 25th–75th percentile. MICs were analyzed on the log_2_ scale; MAR proportions were logit-transformed. Trajectories (MIC, MAR, growth, biofilm) were modeled with linear mixed-effects (fixed: passage/condition; random: lineage nested in founder; restricted maximum likelihood [REML]). Time to high-level resistance was evaluated by Kaplan–Meier with log-rank tests and Cox sensitivity models with founder frailty (extinct lineages censored). Before/after comparisons used paired *t*-tests or Wilcoxon signed-rank tests; effect sizes were reported. Categorical shifts (S/I/R conversion, co-resistance) used McNemar or Fisher’s exact tests. Multiple testing was controlled by Benjamini–Hochberg false-discovery rate (FDR), applied to prespecified families of comparisons (agent-level S → R conversions and groupwise ΔMAR). Software versions were recorded (IBM SPSS Statistics 31, GraphPad Prism 10.6.1, R 4.5.1). Outliers were removed by prespecified QC rules; missing data were not imputed.

## 3. Results

### 3.1. Isolate Recovery, Identification, and Quality Control

**Figure 1:**
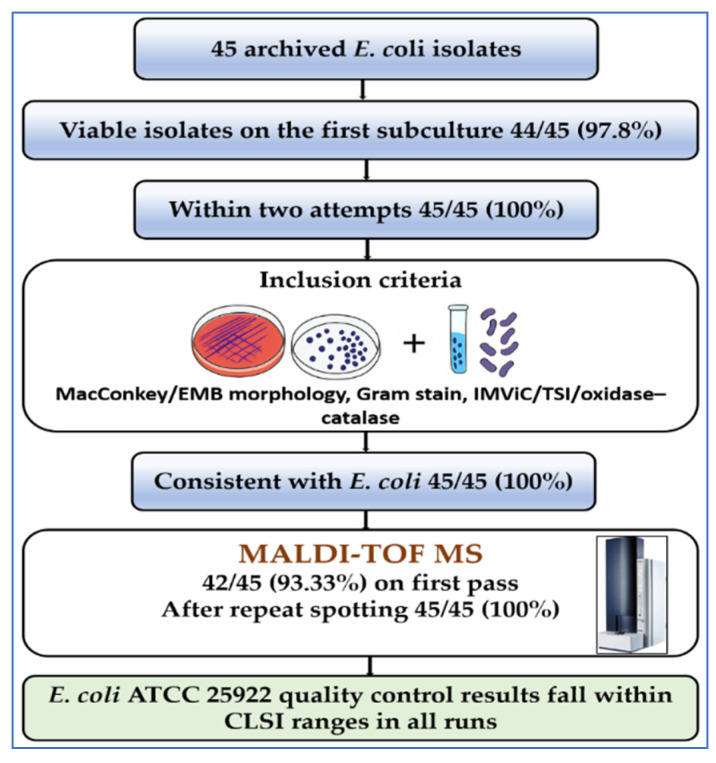
Workflow of isolate recovery, identification, and QC. Schematic showing revival of 45 archived *E. coli* isolates, inclusion screening (MacConkey/EMB morphology, Gram stain, IMViC/TSI, oxidase–catalase), MALDI-TOF MS species confirmation, and QC with *E. coli* ATCC 25922. All procedures were performed in BSL-2 with CLSI M100 (2025) standards. Software: Created in PowerPoint (Microsoft Office 365, v. 2024) and BioRender (v. 2024); labels finalized in GraphPad Prism (v. 10.1.2).

Of 45 archived *E. coli* isolates, 44/45 (97.8%) were viable on first subculture and 45/45 (100%) within two attempts; all met inclusion criteria (Figure 1). Conventional phenotypes were concordant with *E. coli* for all 45/45: lactose-fermenting colonies on MacConkey agar; dark colonies with a metallic-green sheen on EMB agar (when used); Gram-negative rods on Gram stain; an IMViC profile typical of *E. coli* with TSI reactions of acid/acid, gas, and H_2_S-negative; and the expected oxidase-negative/catalase-positive pattern. MALDI-TOF MS yielded log(score) ≥ 2.0 on first pass for 42/45 (93.3%) (median, 2.39; interquartile range [IQR], 2.31–2.47); the remaining three reached ≥ 2.0 after repeat spotting with on-plate formic-acid extraction, resulting in concordant duplicate identifications for 45/45 (100%). No mixed cultures were detected at enrollment. QC with *E. coli* ATCC 25922 fell within CLSI performance ranges in all runs.

**Figure 2:**
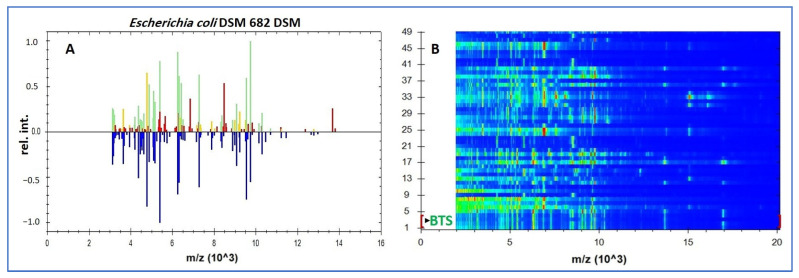
MALDI-TOF MS identification of archived *E. coli*. (**A**) Bruker Biotyper peak-matching profile (~2–16 kDa; x-axis: *m*/*z* (×10*^3^*); y-axis: relative intensity) for a study isolate against the *E. coli* DSM 682 library reference spectrum (MSP); peaks: green = species-level matches (log(score) ≥ 2.0), yellow = genus-level matches, red = unmatched. (**B**) In silico gel view (2–20 kDa; ordinate: *m*/*z* (×10*^3^*)). Rows 1–4: BTS; *E. coli* extract used for calibration/QC; rows 5–49: the 45 study isolates, whose vertically aligned bands illustrate cohort-level concordance of *E. coli* ribosomal fingerprints. Note: DSM 682 is a library reference used for spectral matching and was not cultured; routine AST/QC used *E. coli* ATCC 25922.

Figure 2A illustrates peak matching for a representative isolate against the *E. coli* DSM 682 library reference spectrum (MSP), while Figure 2B shows the cohort gel view with BTS; *E. coli* lanes used for instrument calibration/QC. In this visualization the reference MSP is plotted in blue (below the baseline), while the test-isolate peaks are plotted above the baseline and color-coded by match class: green = species-level matches (log(score) ≥ 2.0), yellow = genus-level matches (log(score) < 2.0; typically 1.7 –< 2.0), and red = peaks without a library match (mismatched). The predominance of green matches and the scarcity of red mismatches are consistent with a high-confidence species assignment. Panel B presents the in silico gel view for all 45 isolates (lanes 1–45), where vertically aligned bands at the same *m*/*z* positions indicate cohort-level concordance of *E. coli* ribosomal-protein fingerprints, with the expected minor variation in band intensity among strains.

### 3.2. Baseline Susceptibility, ESBL/AmpC, MDR, and MAR (Arm 1)

**Figure 3:**
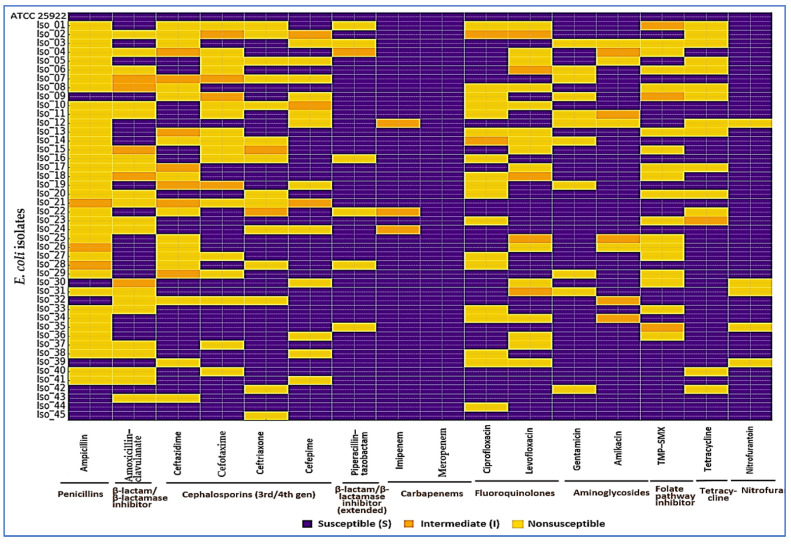
Baseline antimicrobial susceptibility of *E. coli* isolates (Arm 1). Each row is an isolate (top row: ATCC 25922 quality-control strain, all S), and each column is an antibiotic grouped by class. Colors show categorical AST calls per CLSI M100 (2025): purple = susceptible (S), orange = intermediate (I), yellow = nonsusceptible (R). At baseline, nonsusceptibility (%) was: ampicillin 75.6; third-/fourth-generation cephalosporins—cefotaxime 35.6, ceftazidime 33.3, ceftriaxone 31.1, cefepime 26.7; piperacillin–tazobactam 13.3; fluoroquinolones—ciprofloxacin 40.0, levofloxacin 33.3; aminoglycosides—gentamicin 24.4, amikacin 8.9; TMP–SMX 37.8; tetracycline 31.1; nitrofurantoin 11.1; carbapenems—imipenem 0.0, meropenem 0.0. Overall, 28.9% were ESBL-positive, 15.6% AmpC-confirmed, 46.7% met MDR criteria, and the cohort MAR was 0.29 (IQR 0.14–0.50). The control row confirms assay integrity; class grouping makes co-resistance patterns easy to see at a glance.

Founders were stratified to mirror the cohort, so their overall ciprofloxacin MIC median (IQR) matched the 45-isolate set by design: 0.25 µg/mL (0.06–2), with within-group values of 0.06 (0.06–0.125) µg/mL for FQ-S and 1 (0.5–2) µg/mL for LLR. Resistance was highest to ampicillin (34/45, 75.6%), with frequent third-generation cephalosporin nonsusceptibility (cefotaxime 16/45, 35.6%; ceftazidime 15/45, 33.3%; ceftriaxone 14/45, 31.1%; cefepime 12/45, 26.7%). Co-resistance to TMP–SMX (17/45, 37.8%) and tetracycline (14/45, 31.1%) was common, while carbapenem nonsusceptibility was not detected (imipenem 0/45, 0.0%; meropenem 0/45, 0.0%). ESBL phenotype was confirmed in 13/45 (28.9%); 11/45 (24.4%) were cefoxitin screen-positive for AmpC, of which 7/45 (15.6%) met inhibitor-based criteria for AmpC. Nearly half met the MDR definition (≥1 agent in ≥3 classes): 21/45 (46.7%). The cohort MAR index was 0.29 (IQR 0.14–0.50). Consistent with class-level frequencies at baseline—3GCs (26.7–35.6% across agents), TMP–SMX (37.8%), and tetracycline (31.1%) versus carbapenems (0%)—fluoroquinolone-nonsusceptible isolates frequently overlapped with nonsusceptibility in these same classes, as visualized in the heatmap (Figure 3); we therefore refer to these as the predominant co-resistance partners at baseline (Table 1).

### 3.3. Founder Set and Baseline Characteristics (Arm 2 Entry)

To reflect the Arm 1 resistance landscape while enabling parallel evolution, we selected ten founders—five FQ-S and five LLR—balanced by ESBL status, with two independent lineages initiated from each founder (Table 2). Species identity was reconfirmed by MALDI-TOF MS (log(score) ≥ 2.0), and baseline AST was assessed on Vitek 2 and interpreted using CLSI breakpoints, as prespecified in Methods. At entry, FQ-S founders had a median ciprofloxacin MIC of 0.06 µg/mL (IQR 0.06–0.125), whereas LLR founders had 1 µg/mL (IQR 0.5–2); the overall founder-set median was 0.25 µg/mL (IQR 0.06–2). ESBL was detected in 2/5 (40%) FQ-S and 3/5 (60%) LLR founders (5/10, 50% overall). AmpC production was inhibitor-confirmed in 1/5 (20%) of each group (2/10, 20% overall). MDR was observed in 2/5 (40%) FQ-S and 3/5 (60%) LLR founders (5/10, 50% overall). MAR indices were 0.18 (IQR 0.10–0.29) for FQ-S and 0.40 (IQR 0.25–0.55) for LLR founders; overall, 0.29 (IQR 0.14–0.50).

### 3.4. Evolution Under Ciprofloxacin: Trajectories and Time to High-Level Resistance

**Figure 4:**
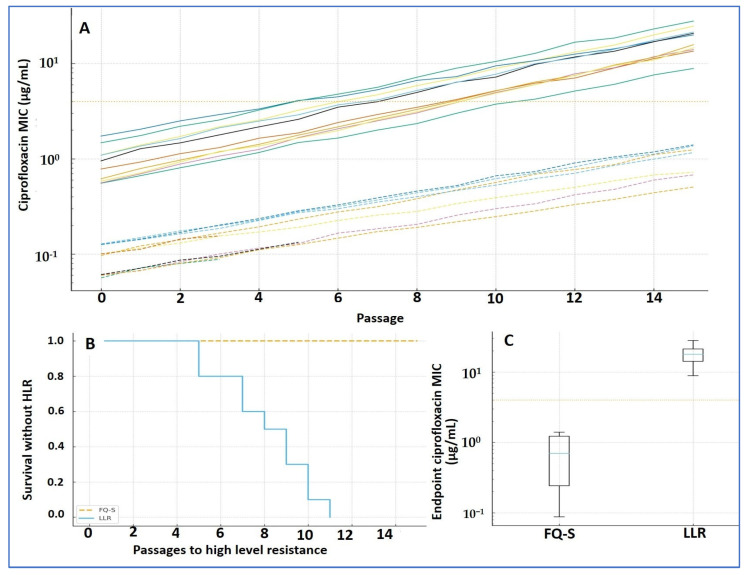
Ciprofloxacin resistance evolution (Arm 2). (**A**) MIC trajectories by passage (log scale) for 20 lineages (two per founder); dashed = FQ-S founders, solid = LLR; dotted line = HLR threshold (MIC ≥ 4 µg/mL). (**B**) Kaplan–Meier time-to-HLR: LLR converts earlier than FQ-S (median 7 vs. 11 passages; log-rank *p* = 0.018). (**C**) Endpoint MICs by founder category (boxplots). Mixed-effects model on log_2_(MIC): passage effect β = 0.22 (95% CI 0.18–0.26; *p* < 0.001); founder effect Δβ = 0.06 (LLR vs. FQ-S; 95% CI 0.02–0.10; *p* = 0.004). Software: R (survival, lme4) and GraphPad.

Key findings. Across 20 parallel lineages, ciprofloxacin MICs rose steadily with passage; 14/20 (70%) crossed the prespecified HLR threshold. Lineages seeded from LLR founders reached HLR earlier (median, 7 passages) than those from FQ-S founders (median, 11 passages). Three FQ-S lineages extinguished before achieving HLR. Trajectories (Figure 4A; Table 3). MICs increased monotonically in both founder groups. At the endpoint, the median MIC was 8 µg/mL (IQR, 4–16), representing a 32-fold rise versus baseline (IQR, 8–128). A linear mixed-effects model of log_2_(MIC) confirmed a strong passage effect (β = 0.22 per passage; 95% CI, 0.18–0.26; *p* < 0.001), with an additional founder-category effect consistent with faster gains from LLR backgrounds (Δβ = 0.06 vs. FQ-S; 95% CI, 0.02–0.10; *p* = 0.004).

Incidence and timing of HLR (Figure 4B; Table 3). Overall, 14/20 lineages achieved HLR (cumulative incidence ≈ 68% by passage 15). The proportion was similar in both founder categories (7/10 FQ-S; 7/10 LLR), but timing differed: LLR lineages converted earlier (median, 7 passages; IQR, 6–9) than FQ-S (median, 11 passages; IQR, 9–14); log-rank *p* = 0.018. Three FQ-S lineages failed early (extinction at 2–4× founder MIC) and were removed from the risk set; three LLR lineages did not reach HLR by passage 30 and were right-censored on the Kaplan–Meier curve.

Endpoints (Figure 4C; Table 3). Endpoint MICs were higher in LLR-derived than FQ-S-derived lineages, consistent with the steeper mixed-effects slope and earlier conversion observed in LLR. Display note. In Figure 4, dashed lines denote FQ-S trajectories and solid lines denote LLR; the survival plot is labeled “Survival without HLR,” with tick marks indicating censored lineages.

### 3.5. Collateral Resistance and MAR Dynamics

Key findings. Under ciprofloxacin selection, resistance broadened—most notably to 3GCs, TMP–SMX, and tetracyclines—while carbapenem activity remained stable. MAR rose alongside increasing ciprofloxacin MICs, with larger gains from LLR founders.

**Figure 5:**
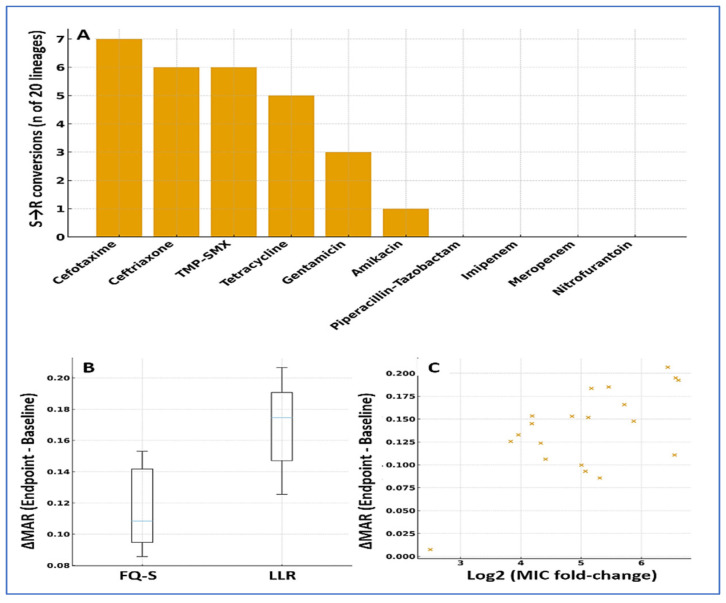
Collateral resistance and MAR dynamics under ciprofloxacin selection. (**A**) Agent-level S → R conversions by endpoint: cefotaxime (7/20), ceftriaxone (6/20), TMP–SMX (6/20), tetracycline (5/20), gentamicin (3/20), amikacin (1/20); carbapenems showed no category shifts. (**B**) ΔMAR by founder category (boxplots): median overall +0.10 (IQR 0.04–0.19); larger in LLR (Fisher’s *p* = 0.047). (**C**) Scatter of ΔMAR versus log_2_ MIC change (Spearman ρ = 0.56; *p* = 0.010). Error bars show median and IQR. Multiple testing: Benjamini–Hochberg FDR. Software: R and GraphPad.

Ciprofloxacin pressure was accompanied by clear collateral shifts in susceptibility (Figure 5). By the endpoint, 12/20 (60%) lineages gained new resistance in at least one additional class, most commonly 3GCs, TMP–SMX, and tetracyclines. At the agent level, S → R conversions were frequent for cefotaxime (7/20, 35%), ceftriaxone (6/20, 30%), TMP–SMX (6/20, 30%), and tetracycline (5/20, 25%); smaller shifts were seen for gentamicin (3/20, 15%) and amikacin (1/20, 5%). In contrast, carbapenems remained stable (0/20 conversions), and piperacillin–tazobactam showed only occasional decreases in susceptibility without consistent category changes.

Overall resistance breadth increased in parallel with ciprofloxacin amplification: the MAR index rose by a median of 0.10 (IQR 0.04–0.19; *p* < 0.001, paired), with 11/20 (55%) lineages showing gains ≥ 0.10. The increases in MAR tracked the rise in ciprofloxacin MICs (Spearman ρ = 0.56; *p* = 0.010), indicating a moderate, monotonic association between resistance level and co-resistance breadth. Collateral expansion was more common in LLR-derived lineages (8/10, 80%) than in FQ-S lineages (4/10, 40%; Fisher’s *p* = 0.047), mirroring the earlier time-to-HLR advantage seen in LLR backgrounds (Table 3; Figure 5).

### 3.6. Growth Fitness and Biofilm Metrics

**Figure 6:**
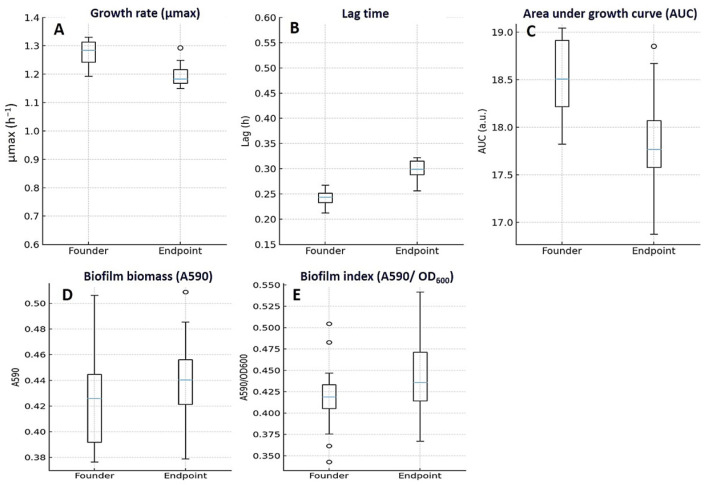
Growth, fitness, and biofilm characteristics of *E. coli* founders and endpoints. Endpoint isolates showed small fitness costs in drug-free medium: maximum specific growth rate (μmax, h^−1^) decreased modestly (**A**), lag time (h) increased (**B**), and overall growth output (AUC, a.u.) declined slightly (**C**) compared with founders. In contrast, biofilm biomass (A590) (**D**) and the Biofilm Index (A590/OD600) (**E**) remained broadly similar. Boxplots show median (center line) and IQR (box); whiskers represent 1.5×IQR; points are outliers; founders n = 10 and endpoints n = 20. Panel medians correspond exactly to Table 4 (e.g., μmax founders 1.28 [1.22–1.34] h^−1^ vs. endpoints 1.19 [1.12–1.25] h^−1^). Raw units are displayed on all axes.

Key findings. Endpoints carried small fitness costs in drug-free medium—slightly slower growth and a longer lag—that were partly offset under subinhibitory ciprofloxacin. Carrying capacity changed very little, and biofilm biomass was broadly similar to that of the founders. Figure 6 displays the same distributions summarized in Table 4 (medians with IQR; whiskers 1.5 × IQR), so plotted medians match tabulated values (e.g., μmax founders 1.28 [1.22–1.34] h^−1^ vs. endpoints 1.19 [1.12–1.25] h^−1^).

In antibiotic-free medium, endpoint lineages grew a bit more slowly than their founders (Figure 6A–C; Table 4). The maximum specific growth rate fell from 1.28 h^−1^ (IQR 1.22–1.34) at baseline to 1.19 h^−1^ (1.12–1.25) at endpoint (median Δ −0.08; IQR −0.16 to −0.02; *p* = 0.030), and lag time lengthened from 0.24 h (0.22–0.27) to 0.30 h (0.27–0.34) (median Δ +0.06; 0.03–0.09; *p* = 0.020). Carrying capacity was essentially unchanged (0.93 OD_600_ [0.89–0.97] → 0.92 [0.88–0.96]; Δ −0.01; −0.04 to +0.01; *p* = 0.190), while the overall AUC dipped modestly (18.7 AU [17.9–19.3] → 17.9 [17.1–18.7]; Δ −0.8; −1.5 to −0.2; *p* = 0.030). These patterns appeared in both founder categories but were clearer in FQ-S–derived lineages (e.g., μmax Δ −0.12; paired Wilcoxon *p* = 0.020; AUC Δ −1.1; *p* = 0.020). In contrast, within LLR-derived lineages, the paired changes were small and not statistically significant—μmax Δ −0.05 (*p* = 0.090), lag time Δ +0.05 h (*p* = 0.060), carrying capacity Δ −0.01 OD_600_ (*p* = 0.410), and AUC Δ −0.4 AU (*p* = 0.180).

Under sub-inhibitory ciprofloxacin, performance improved: overall AUC rose from 12.7 AU (12.0–13.6) to 13.5 (12.7–14.3) (median Δ +0.7; 0.1–1.4; *p* = 0.030), suggesting a context-dependent compensation of drug-free fitness costs. Biofilm biomass remained largely stable from founders to endpoints (0.42 OD_590_ [0.39–0.47] → 0.45 [0.40–0.49]; Δ +0.02; −0.01 to +0.04; *p* ns), and there was no group effect at endpoint (FQ-S vs. LLR, *p* = 0.62) (Figure 6D,E). This gain reached significance in FQ-S lineages (AUC Δ +0.9 AU; *p* = 0.040), but not in LLR lineages (AUC Δ +0.6 AU; *p* = 0.070).

## 4. Discussion

FQ resistance in *E. coli* continues to narrow empiric options and often co-occurs with resistance to key oral alternatives. WHO BPPL 2024 and GLASS highlight 3GC-resistant *E. coli* as a sustained public-health threat with marked regional heterogeneity, consistent with 2019 AMR burden estimates and national summaries (e.g., English Surveillance Program for Antimicrobial Utilization and Resistance [ESPAUR] 2023–2024) showing high FQ and 3GC resistance with preserved carbapenem activity [33,34,35]. For program benchmarking, WHO/Our World in Data (OWID) dashboards track the share of *E. coli* bloodstream infections resistant to 3GCs. In this context, a phenotype-first, sequencing-independent framework—MALDI-TOF MS identification, automated AST with CLSI criteria, and tractable indices such as MAR—offers an immediately deployable option for labs without routine WGS, without re-stating methods [21,36,37].

Viewed through the lens of our results, the value of a sequencing-independent, routine-laboratory framework is chiefly practical: it allows teams to follow ciprofloxacin MIC trajectories and time-to-HLR, track MAR alongside collateral bundles, and read simple fitness/biofilm changes—all without WGS. In this cohort, earlier HLR from LLR founders, MAR increases that paralleled MIC amplification, and preserved carbapenem activity together yield usable stewardship signals: avoid empiric FQs where local nonsusceptibility approaches ~40%, be cautious about cycling directly into 3GCs/TMP–SMX/tetracyclines after FQs, prioritize early AST-guided de-escalation, and flag LLR isolates as high-risk for rapid conversion to HLR. Because these readouts are CLSI-aligned and QC-anchored, they are portable across laboratories; targeted genotyping (QRDR, PMQR, efflux/porin expression) can be layered later to verify mechanisms without changing the immediate clinical guidance.

Across phenotype-only clinical cohorts, FQ-nonsusceptible *E. coli* frequently co-resist ampicillin and TMP–SMX, while carbapenem susceptibility is generally preserved [38]—a pattern directionally concordant with our baseline profile [39,40]. Emergency department and outpatient urinary series consistently report high ampicillin and substantial TMP–SMX resistance among *E. coli* urine isolates, alongside double-digit FQ resistance [40,41]. Multiple hospital and surveillance summaries likewise emphasize that carbapenem resistance remains relatively low compared with elevated FQ resistance, reinforcing the observed preservation of carbapenem activity [39].

In Arm 1, ciprofloxacin and levofloxacin nonsusceptibility reached 40.0% and 33.3%, respectively; ESBL was confirmed in 28.9%, AmpC in 15.6%, nearly half of the isolates were MDR (46.7%), and the cohort MAR index was 0.29 (IQR 0.14–0.50). These rates mirror recent multi-setting summaries and align with the mechanistic landscape—target-site mutations, efflux upregulation, and decreased permeability—described in contemporary reviews [6,7]. In Arm 2, 70% of lineages achieved HLR under stepwise ciprofloxacin; conversion occurred earlier from LLR founders than from FQ-S founders. Collateral resistance accrued most often to 3GCs, TMP–SMX, and tetracyclines, while carbapenem susceptibility remained stable. Notably, MALDI-TOF identifications met conventional performance thresholds (species-level log(score) ≥ 2.0), consistent with large clinical series [36]. Fitness costs in a drug-free medium were modest (lower μmax, longer lag), and biofilm biomass changed little. These readouts provide a deployable map of resistance trajectories, collateral effects, and fitness trade-offs that routine laboratories can apply to anticipate stewardship risks.

Clinical series of complicated UTIs demonstrate high FQ resistance in *E. coli* and the prominence of successful clonal groups (e.g., ST131), capturing real-world selective pressures but not resolving time-to-event amplification [42]. In contrast, in vitro evolution experiments show rapid, stepwise ciprofloxacin MIC increases and collateral effects under controlled exposure—establishing phenotypic trajectories that align with our framework’s time-to-HLR and ΔMAR readouts [12,43]. Within-host work similarly shows that ciprofloxacin exposure can select and expand FQ-resistant *E. coli* in the gut [44]. These clinical and in vitro comparators support external validity (co-resistance patterns; preserved carbapenem activity) while highlighting the added value of explicit time-to-HLR endpoints and ΔMAR-summarized collateral bundles for routine laboratories [12,42,43].

What is unique here is a decision-oriented structure—parallel lineages, a prespecified time-to-HLR endpoint, ΔMAR-based collateral bundles, and explicit stewardship mapping—implemented without sequencing to convert routine phenotypes into risk-of-amplification and co-selection signals [45]. Multiple external lines of evidence are consistent with our observations. At the clinical population level, double-digit FQ nonsusceptibility and convergence with ESBL/MDR patterns across community and healthcare settings mirror prior syntheses [5,7], while GLASS analyses and the WHO BPPL 2024 emphasize high burdens of 3GC-resistant *E. coli* with substantial regional heterogeneity [2,4]. Updated population estimates (Global Research on Antimicrobial Resistance [GRAM] 2024) reinforce the scale of the problem, and ESPAUR reports document persistent FQ nonsusceptibility alongside stable carbapenem susceptibility [34]. In evolution studies, rapid, stepwise increases in ciprofloxacin MICs with predictable trajectories are well documented and typically initiated by mutations in the QRDR, followed by adjunct mechanisms [43,46]. Experimental and clinical genetics consistently highlight canonical QRDR paths—*gyrA* S83L/D87N and *parC* S80I—followed by secondary changes and global regulators [47]. Plasmid-mediated quinolone resistance (PMQR) elements (e.g., *qnr*, *aac(6′)-Ib-cr*, *qepA*/*oqxAB*) can provide LLR that accelerates selection of high-level QRDR mutations under FQ exposure [48]. PMQR elements (e.g., *qnr*, *aac*(6′)-Ib-cr, *qepA*/*oqxAB*) can provide LLR that accelerates selection of high-level QRDR mutations under FQ exposure [48]. Our collateral bundle is coherent with upregulated AcrAB–TolC and decreased permeability that broaden resistance beyond target mutations [49,50]; efflux-dominated cross-resistance and *mar*/*sox*/*rob*-regulated envelope remodeling are repeatedly implicated in broadening MAR while leaving carbapenem activity relatively preserved [47,51]. The modest growth penalties we observed align with theory and meta-analyses showing small but measurable fitness costs and partial compensation over time [10,52], and in vitro work indicates that drug level, exposure schedule, and genetic background modulate both these costs and the trajectory to HLR [47]. Clinically, preserved carbapenem activity amid rising MAR aligns with guidance that carbapenems remain reliable for serious ESBL-producing *E. coli* infections even as oral options narrow [1]; current Infectious Diseases Society of America (IDSA) guidance recommends a carbapenem as first-line therapy for ESBL-producing Enterobacterales infections outside the urinary tract [53].

Important contrasts also warrant consideration. Several mapping studies report robust collateral sensitivity (CS) that can be leveraged for cycling [54,55,56], whereas other work shows that CS effects can depend on evolutionary paths and genetic background and may erode into cross-resistance [57,58]. Foundational *E. coli* studies proposed CS-based cycling to avoid resistance, but subsequent theory and higher-throughput screens show that CS is not universal and depends on repeatable trajectories and strain background [55,57,59]. Thus, while CS-informed cycling is promising, implementation should be anchored to local genotype–phenotype data to avoid inadvertently selecting cross-resistance [59]. For biofilm phenotypes, sustained ciprofloxacin exposure can enrich resistance and remodel biofilm properties in specific models [12,60], yet we observed little change in biomass—underscoring dependence on exposure schedule, founder genotype, and ecology. Recent work indicates that biofilm-resident *E. coli* exposed to high ciprofloxacin can follow canonical target-mutation routes to HLR, whereas biomass responses at sub-MICs vary; our neutral biomass shift is therefore within expected heterogeneity [12].

Mechanistically, earlier conversion to HLR among LLR founders supports a shorter-path model in which prior target alterations and/or primed global tolerance states (e.g., *mar*/*sox*/*rob*-regulated AcrAB–TolC activation, decreased permeability) reduce the steps required to cross the HLR threshold under ciprofloxacin. The observed collateral bundle (3GCs, TMP–SMX, tetracyclines) is consistent with efflux-dominated amplification that inflates MAR without affecting carbapenems [8,46,49,61]. Literature supports three testable predictions for our endpoints: (i) early QRDR steps (*gyrA*/*parC*) in fast-rising lineages; (ii) *marA*/*soxS*/*rob* perturbations and porin changes tracking ΔMAR; and (iii) partial MIC reversal with an RND efflux inhibitor such as phenyl-arginine-β-naphthylamide (PAβN), consistent with efflux involvement [47,62]. These predictions outline a feasible roadmap for confirmation (QRDR sequencing; efflux/porin expression; PAβN modulation assays) [51,62].

Reconciling resistance gain with fitness costs. Although LLR-derived lineages reached high-level ciprofloxacin resistance sooner than FQ-S and broadened collateral resistance, their drug-free fitness losses were modest and often not statistically significant. In contrast, FQ-S endpoints showed slightly larger drops in μmax and AUC and contributed all three early extinctions before HLR. A simple explanation is starting-point asymmetry: LLR founders likely already carry tolerance/resistance determinants and prior compensatory adjustments, so the marginal cost of additional steps to HLR is smaller; by comparison, FQ-S founders must first acquire costlier target or regulatory changes, with compensation lagging behind [10,52,63]. This interpretation aligns with evidence that fitness effects are highly background-dependent and can be mitigated by compensation, and that some quinolone routes—e.g., common *gyrA* paths—can yield high resistance with comparatively limited fitness penalty [64,65]. In our data, only FQ-S lineages failed early at low fold-increases over baseline MIC, whereas the overall proportion reaching HLR was equal (7/10 per group)—indicating that the primary difference is one of timing and fitness trajectory, not ultimate success.

From a stewardship perspective, the Arm 1 landscape (FQ nonsusceptibility 33–40%, ESBL 28.9%, MDR 46.7%, MAR median 0.29) makes routine empiric FQ use difficult to justify in our setting; early transition to targeted therapy once AST is available is advisable [66]. Because the collateral bundle concentrates in 3GCs, TMP–SMX, and tetracyclines, cycling immediately into these same classes after FQ exposure risks therapeutic erosion, consistent with mechanistic links to efflux and permeability [49]. Conversely, guidance supports carbapenem escalation for severe ESBL-producing Enterobacterales infections when indicated, reserving narrower or oral agents for de-escalation when susceptibility allows [53]. Programmatically, MAR offers a practical co-selection indicator for stewardship dashboards, and MALDI-TOF species-level calls (log(score) ≥ 2.0) are sufficient for rapid, reproducible identification workflows across laboratories [32,36]. Together with the updated WHO priority list and GLASS heterogeneity, these data justify prioritizing local FQ-resistance surveillance, MAR tracking, and early confirmation testing. Baseline LLR can serve as a risk marker for rapid HLR under continued FQ pressure, and cohort-level MAR is a tractable metric for following co-selection over time; these inferences align with WHO 2024 priorities, GLASS patterns, and IDSA guidance [67].

Finally, our findings fit broader dynamics observed in controlled evolution systems: *E. coli* typically acquires resistance stepwise, with rising MICs driven first by QRDR substitutions (*gyrA*/*parC*) and then by adjunct mechanisms—a pattern shown in morbidostat and time-series “resistomics” studies that resolve shared versus lineage-specific trajectories [43,46]. Adjunct changes plausibly include *mar*/*sox*/*rob*-mediated stress responses, porin downregulation/loss, and plasmid-mediated determinants that raise the MIC “floor,” thereby reducing the steps required to cross the HLR threshold [8]. The collateral bundle observed here (3GCs, TMP–SMX, tetracyclines) is mechanistically coherent with AcrAB–TolC upregulation and decreased outer-membrane permeability, both of which broaden resistance beyond target-site change [49,50]. These interpretations yield specific, testable predictions for our endpoints—earlier/more frequent *gyrA*/*parC* steps in LLR-derived lineages; *acrB*/*tolC* overexpression or *marR*/*soxR*/*rob* perturbations tracking ΔMAR and collateral breadth; partial MIC reduction with PAβN in collateral-rich endpoints; and modulation of effect size (but not direction) by ESBL/AmpC background [68].

Clinical implications. In a setting where *E. coli* FQ nonsusceptibility is ~40%, empiric FQs are unreliable and should be replaced by agents guided by local data and rapid susceptibility testing. Stewardship should prioritize early de-escalation once AST results are available and track MAR as a practical co-selection signal, since rising MAR paralleled ciprofloxacin MIC amplification in this cohort. Given the observed collateral bundle, clinicians should avoid cycling directly from FQs into 3GCs, TMP–SMX, or tetracyclines without confirmed activity. Carbapenems remain an appropriate safeguard for severe ESBL-producing infections, with de-escalation to narrower active options when susceptibility permits. Finally, isolates with LLR should be considered at high risk for rapid conversion to HLR under continued exposure, warranting targeted therapy choices and proactive stewardship oversight.

## 5. Strengths and Limitations of the Study

This sequencing-independent, phenotype-first design is readily deployable in routine laboratories, pairing standardized identification/AST (MALDI-TOF MS; automated AST with CLSI M100 interpretation) and rigorous QC with explicit quantification of ESBL/AmpC, MDR, and MAR indices, plus a replicated, parallel-lineage evolution model that enabled trajectory modeling and time-to-HLR analysis. The framework yields stewardship-relevant signals (e.g., rising MAR tracking ciprofloxacin MIC amplification and faster HLR conversion from LLR founders) while using reproducible methods and prespecified endpoints. Limitations include a single-center cohort and modest sample size (45 isolates, 10 founders, 20 lineages); in vitro batch selection rather than morbidostat or host-mimicking media; absence of WGS/targeted genotyping (mechanisms inferred); and a biofilm readout focused on biomass rather than structure/tolerance. To confirm why the resistance patterns we observed occur, we will add focused mechanistic work: targeted QRDR sequencing (*gyrA*/*gyrB*, *parC*/*parE*), screening for PMQR determinants (*qnrA*/*qnrB*/*qnrS*, *aac*(6′)-Ib-cr, *qepA*, *oqxAB*), and efflux/permeability assays (PAβN modulation; *tolC* and porin expression), alongside transcriptional profiling of *acrA*/*acrB* and *mar*/*sox*/*rob* regulators. Where category definitions matter (e.g., MDR), we applied internationally harmonized criteria to keep interpretations consistent and comparable.

## 6. Future Directions

In practical terms, we aim to show how resistance arises and turn that insight into usable clinical choices. First, we will directly link genotype to phenotype by sequencing QRDR and key efflux-regulator loci (e.g., *marR*/*soxR*/*rob*) in founders and endpoints, paired with efflux activity assays (±inhibitor) to quantify how AcrAB–TolC shapes resistance trajectories and collateral patterns. Second, we will systematically map collateral responses in founders and endpoints to pinpoint stable, clinically usable collateral sensitivities for rotation or combination therapy, checking that they hold across passages and genetic backgrounds. Third, we will test robustness under host-like conditions (e.g., urine matrices, low pH, microaerobic/anaerobic states) and during biofilm-adapted selection, adding endpoints that capture tolerance and community architecture—not just biomass. Finally, to support clinical translation, we will integrate these trajectories with PK/PD (time–kill studies and exposure–response modeling) so inferred selection windows align with achievable drug levels and can inform regimen design, de-escalation strategies, and stewardship policy.

## 7. Conclusions

This study demonstrates that a phenotype-first strategy, anchored in routine clinical microbiology tools, can generate reproducible and clinically actionable insights into FQ resistance dynamics in *E. coli*. We showed that ciprofloxacin nonsusceptibility was already high (40%) in archived isolates, with frequent ESBL/AmpC phenotypes and nearly half of the strains meeting the MDR definition. Under controlled ciprofloxacin exposure, 70% of lineages rapidly progressed to high-level resistance, and low-level resistant founders converted significantly earlier than fully susceptible ones. Collateral resistance was most pronounced to third-generation cephalosporins, trimethoprim–sulfamethoxazole, and tetracyclines, while carbapenem susceptibility was preserved. Beyond experimental findings, this framework has direct translational relevance. Because it relies on accessible platforms—MALDI-TOF MS, CLSI-guided AST, MAR index tracking, and classical phenotypic assays—it can be readily implemented in routine laboratories, even where sequencing is not available. The MAR index in particular emerges as a practical co-selection indicator for stewardship dashboards, allowing hospitals to anticipate collateral resistance risks in real time. Clinically, our results argue against empirical FQ use in high-resistance settings and support early switch to targeted therapy once AST results are available, while also reinforcing carbapenems as a safeguarded option for severe ESBL-producing infections. In summary, our findings underscore two key risks—the rapid amplification of FQ resistance and the parallel expansion of MDR through efflux-linked collateral effects—but also highlight practical stewardship opportunities. This phenotype-first, reproducible approach complements genomics-based surveillance and offers a scalable model for linking laboratory data to antimicrobial stewardship in diverse healthcare settings.

## Figures and Tables

**Table 1:** Baseline antimicrobial susceptibility profiles, ESBL/AmpC phenotypes, MDR prevalence, and MAR indices of 45 archived *E. coli* isolates (Arm 1).

Antimicrobial Class	Agent(s) Tested	Nonsusceptible	
		Number of Isolates	Percentage
Penicillins	Ampicillin	34/45	75.6
β-lactam/β-lactamase inhibitor	Amoxicillin–clavulanate	19/45	42.2
Cephalosporins (3rd/4th gen)	Cefotaxime	16/45	35.6
	Ceftazidime	15/45	33.3
	Ceftriaxone	14/45	31.1
	Cefepime	12/45	26.7
β-lactam/β-lactamase inhibitor (extended)	Piperacillin–tazobactam	6/45	13.3
Carbapenems	Imipenem	0/45	0.0
	Meropenem	0/45	0.0
Fluoroquinolones	Ciprofloxacin	18/45	40.0
	Levofloxacin	15/45	33.3
Aminoglycosides	Gentamicin	11/45	24.4
	Amikacin	4/45	8.9
Folate pathway inhibitor	TMP–SMX	17/45	37.8
Tetracycline class	Tetracycline	14/45	31.1
Nitrofuran	Nitrofurantoin	5/45	11.1
Resistance phenotypes	ESBL confirmed	13/45	28.9
	AmpC screen-positive (cefoxitin)	11/45	24.4
	— inhibitor-based AmpC confirmed	7/45	15.6
Composite indices	MDR prevalence (≥1 agent in ≥3 classes)	21/45	46.7
	MAR index, median (IQR) [value: 0.29 (0.14–0.50)]	—	—
Frequent co-resistance partners	Third-generation cephalosporins; TMP–SMX; tetracyclines	—	—

**Table 2:** The founder set composition and baseline characteristics (Arm 2 entry).

Founder Group	No.	Ciprofloxacin MIC, Median (IQR) (µg/mL)	ESBL Positive		AmpC Confirmed		MDR		MAR Index, Median (IQR)
			No.	%	No.	%	No.	%	
FQ-S	5	0.06 (0.06–0.125)	2	40%	1	20%	2	40%	0.18 (0.10–0.29)
LLR	5	1 (0.5–2)	3	60%	1	20%	3	60%	0.40 (0.25–0.55)
Overall	10	0.25 (0.06–2)	5	50%	2	20%	5	50%	0.29 (0.14–0.50)

Note: Founders were deliberately stratified by FQ susceptibility to preserve the cohort’s baseline MIC distribution; therefore, the overall founder median/IQR equals that of the full 45-isolate set, while FQ-S and LLR rows show the intended separation.

**Table 3:** Evolution under ciprofloxacin (Arm 2): trajectories and time to high-level resistance (HLR).

Outcome/Metric	Overall 10 Founders; 20 Lineages	FQ-S Lineages 5 Founders; 10 Lineages	LLR Lineages 5 Founders; 10 Lineages	Statistics/Notes
Reached HLR, n/N (%)	14/20 (70%)	7/10 (70%)	7/10 (70%)	Kaplan–Meier cumulative incidence at passage 15 ≈ 68%
Passages to HLR, median (IQR)	NE ^1^	11 (9–14)	7 (6–9)	Log-rank *p* = 0.018 (LLR earlier than FQ-S)
Lineage extinction before HLR, n/N (%)	3/20 (15%)	3/10 (30%)	0/10 (0%)	Failures at 2–4× founder MIC
Endpoint ciprofloxacin MIC (μg/mL), median (IQR)	8 (4–16)	NR ^2^	NR ^2^	Median among surviving lineages at endpoint
MIC fold-increase vs. founder, median (IQR)	32× (8–128×)	NR ^2^	NR ^2^	Median across lineages
Mixed-effects model: passage effect (β on log_2_(MIC))	0.22 per passage	NE ^1^	NE ^1^	95% CI 0.18–0.26; *p* < 0.001
Mixed-effects model: founder effect (LLR vs. FQ-S)	NE ^1^	NE ^1^	NE ^1^	Δβ = 0.06 (95% CI 0.02–0.10); *p* = 0.004

Footnotes: ^1^ NE, not estimated at the overall level because differing censoring/extinction patterns between groups make a single pooled median time-to-event non-informative; group medians are reported. ^2^ NR, not reported at the subgroup level to avoid redundancy with Figure 4C (boxplots show the full distribution); the overall endpoint MIC is provided here for numerical reference.

**Table 4:** Growth, fitness, and biofilm metrics (Arm 2).

Category	Metric	Group	Founders Median (IQR) [n]	Endpoints Median (IQR) [n]	Delta Endpoint–Founder Median (IQR)	Notes/*p*-Value
Drug-free growth	µmax, h^−1^	FQ-S	1.30 (1.22–1.36) [n = 5]	1.18 (1.12–1.24) [n = 10]	−0.12 (−0.20 to −0.05)	paired Wilcoxon *p* = 0.020
		LLR	1.25 (1.18–1.32) [n = 5]	1.20 (1.12–1.26) [n = 10]	−0.05 (−0.12 to 0.00)	paired Wilcoxon *p* = 0.090
		Overall	1.28 (1.22–1.34) [n = 10]	1.19 (1.12–1.25) [n = 20]	−0.08 (−0.16 to −0.02)	paired Wilcoxon *p* = 0.030
	Lag time (h)	FQ-S	0.25 (0.22–0.28) [n = 5]	0.32 (0.28–0.36) [n = 10]	+0.07 (+0.04 to +0.10)	paired Wilcoxon *p* = 0.010
		LLR	0.24 (0.21–0.27) [n = 5]	0.29 (0.26–0.33) [n = 10]	+0.05 (+0.02 to +0.08)	paired Wilcoxon *p* = 0.060
		Overall	0.24 (0.22–0.27) [n = 10]	0.30 (0.27–0.34) [n = 20]	+0.06 (+0.03 to +0.09)	paired Wilcoxon *p* = 0.020
	Carrying capacity (OD_600_)	FQ-S	0.92 (0.88–0.96) [n = 5]	0.90 (0.86–0.95) [n = 10]	−0.02 (−0.05 to +0.01)	paired Wilcoxon *p* = 0.280
		LLR	0.94 (0.90–0.98) [n = 5]	0.93 (0.89–0.97) [n = 10]	−0.01 (−0.04 to +0.02)	paired Wilcoxon *p* = 0.410
		Overall	0.93 (0.89–0.97) [n = 10]	0.92 (0.88–0.96) [n = 20]	−0.01 (−0.04 to +0.01)	paired Wilcoxon *p* = 0.190
	AUC, AU	FQ-S	18.5 (17.8–19.2) [n = 5]	17.4 (16.5–18.2) [n = 10]	−1.1 (−1.8 to −0.4)	paired Wilcoxon *p* = 0.020
		LLR	18.8 (18.0–19.5) [n = 5]	18.4 (17.6–19.0) [n = 10]	−0.4 (−1.2 to +0.3)	paired Wilcoxon *p* = 0.180
		Overall	18.7 (17.9–19.3) [n = 10]	17.9 (17.1–18.7) [n = 20]	−0.8 (−1.5 to −0.2)	paired Wilcoxon *p* = 0.030
Sub-inhibitory ciprofloxacin	AUC, AU	FQ-S	12.0 (11.3–12.8) [n = 5]	12.9 (12.1–13.7) [n = 10]	+0.9 (+0.2 to +1.6)	paired Wilcoxon *p* = 0.040
		LLR	13.4 (12.6–14.1) [n = 5]	14.0 (13.3–14.7) [n = 10]	+0.6 (0.0 to +1.3)	paired Wilcoxon *p* = 0.070
		Overall	12.7 (12.0–13.6) [n = 10]	13.5 (12.7–14.3) [n = 20]	+0.7 (+0.1 to +1.4)	paired Wilcoxon *p* = 0.030
Biofilm phenotype	Crystal violet biomass (OD_590_)	FQ-S	0.42 (0.38–0.46) [n = 5]	0.44 (0.39–0.48) [n = 10]	+0.02 (−0.01 to +0.04)	group effect (endpoint) FQ-S vs. LLR: *p* = 0.62
		LLR	0.43 (0.40–0.47) [n = 5]	0.45 (0.41–0.49) [n = 10]	+0.02 (−0.01 to +0.05)	group effect (endpoint) FQ-S vs. LLR: *p* = 0.62
		Overall	0.42 (0.39–0.47) [n = 10]	0.45 (0.40–0.49) [n = 20]	+0.02 (−0.01 to +0.04)	no systematic shift across lineages

## Data Availability

The raw data supporting the conclusions of this article will be made available by the authors on request.

## References

[1] Tamma P.D., Heil E.L., Justo J.A., Mathers A.J., Satlin M.J., Bonomo R.A. (2024). Infectious Diseases Society of America 2024 guidance on the treatment of antimicrobial-resistant gram-negative infections. Clin. Infect. Dis..

[2] Ajulo S., Awosile B. (2024). Global antimicrobial resistance and use surveillance system (GLASS 2022): Investigating the relationship between antimicrobial resistance and antimicrobial consumption data across the participating countries. PLoS ONE.

[3] Cuevas O., Oteo J., Lazaro E., Aracil B., De Abajo F., Garcia-Cobos S., Ortega A., Campos J., Group S.E.-N.S., Fontanals D. (2011). Significant ecological impact on the progression of fluoroquinolone resistance in *Escherichia coli* with increased community use of moxifloxacin, levofloxacin and amoxicillin/clavulanic acid. J. Antimicrob. Chemother..

[4] WHO (2024). WHO Bacterial Priority Pathogens List, 2024: Bacterial Pathogens of Public Health Importance to Guide Research, Development and Strategies to Prevent and Control Antimicrobial Resistance.

[5] Stapleton A.E., Wagenlehner F.M., Mulgirigama A., Twynholm M. (2020). *Escherichia coli* resistance to fluoroquinolones in community-acquired uncomplicated urinary tract infection in women: A systematic review. Antimicrob. Agents Chemother..

[6] WHO (2022). Global Antimicrobial Resistance and Use Surveillance System (GLASS) Report: 2022.

[7] Ruiz-Lievano A.P., Cervantes-Flores F., Nava-Torres A., Carbajal-Morales P.J., Villaseñor-Garcia L.F., Zavala-Cerna M.G. (2024). Fluoroquinolone resistance in *Escherichia coli* causing community-acquired urinary tract infections: A systematic review. Microorganisms.

[8] Hooper D.C., Jacoby G.A. (2015). Mechanisms of drug resistance: Quinolone resistance. Ann. N. Y. Acad. Sci..

[9] Strahilevitz J., Jacoby G.A., Hooper D.C., Robicsek A. (2009). Plasmid-mediated quinolone resistance: A multifaceted threat. Clin. Microbiol. Rev..

[10] Andersson D.I., Hughes D. (2010). Antibiotic resistance and its cost: Is it possible to reverse resistance?. Nat. Rev. Microbiol..

[11] Byrd B.A., Zenick B., Rocha-Granados M.C., Englander H.E., Hare P.J., LaGree T.J., DeMarco A.M., Mok W.W. (2021). The AcrAB-TolC efflux pump impacts persistence and resistance development in stationary-phase *Escherichia coli* following delafloxacin treatment. Antimicrob. Agents Chemother..

[12] Nesse L.L., Osland A.M., Asal B., Mo S.S. (2023). Evolution of antimicrobial resistance in *E. coli* biofilm treated with high doses of ciprofloxacin. Front. Microbiol..

[13] Wilson C., Lukowicz R., Merchant S., Valquier-Flynn H., Caballero J., Sandoval J., Okuom M., Huber C., Brooks T.D., Wilson E. (2017). Quantitative and qualitative assessment methods for biofilm growth: A mini-review. Res. Rev. J. Eng. Technol..

[14] Pitout J.D., Peirano G., Chen L., DeVinney R., Matsumura Y. (2022). *Escherichia coli* ST1193: Following in the footsteps of *E. coli* ST131. Antimicrob. Agents Chemother..

[15] Reid C.J., McKinnon J., Djordjevic S.P. (2019). Clonal ST131-H 22 *Escherichia coli* strains from a healthy pig and a human urinary tract infection carry highly similar resistance and virulence plasmids. Microb. Genom..

[16] Muloi D.M., Jauneikaite E., Anjum M.F., Essack S.Y., Singleton D.A., Kasudi M.R., Wade M.J., Egyir B., Nunn J.G., Midega J.T. (2023). Exploiting genomics for antimicrobial resistance surveillance at One Health interfaces. Lancet Microbe.

[17] Sundermann A.J., Rosa R., Harris P.N., Snitkin E., Javaid W., Moore N.M., Hayden M.K., Allen K., Rodino K., Peacock S.J. (2025). Pathogen genomics in healthcare: Overcoming barriers to proactive surveillance. Antimicrob. Agents Chemother..

[18] Teichmann L., Luitwieler S., Bengtsson-Palme J., Ter Kuile B. (2025). Fluoroquinolone-specific resistance trajectories in *E. coli* and their dependence on the SOS-response. BMC Microbiol..

[19] Dash J., Naykodi A., Mohakud N.K., Deb S. (2024). MALDI TOF-MS for microbial identification and diagnosis. Evolving Landscape of Molecular Diagnostics.

[20] Patel R. (2015). MALDI-TOF MS for the diagnosis of infectious diseases. Clin. Chem..

[21] Elbehiry A., Abalkhail A. (2025). Spectral Precision: Recent Advances in Matrix-Assisted Laser Desorption/Ionization Time-of-Flight Mass Spectrometry for Pathogen Detection and Resistance Profiling. Microorganisms.

[22] Elbehiry A., Marzouk E., Moussa I.M., Alenzi A., Al-Maary K.S., Mubarak A.S., Alshammari H.D., Al-Sarar D., Alsubki R.A., Hemeg H.A. (2021). Multidrug-resistant *Escherichia coli* in Raw Milk: Molecular Characterization and the potential impact of camel’s Urine as an Antibacterial Agent. Saudi J. Biol. Sci..

[23] Papadomanolaki A., Siopi M., Karakosta P., Vourli S., Pournaras S. (2022). Comparative Evaluation of Vitek 2 and Etest versus Broth microdilution for ceftazidime/avibactam and ceftolozane/tazobactam susceptibility testing of *Enterobacterales* and *Pseudomonas aeruginosa*. Antibiotics.

[24] Weinstein M.P., Limbago B., Patel J., Mathers A., Campeau S., Mazzulli T., Eliopoulos G., Patel R., Galas M., Richter S. (2018). M100 performance standards for antimicrobial susceptibility testing. Clin. Lab. Stand. Inst..

[25] Wiegand I., Hilpert K., Hancock R.E. (2008). Agar and broth dilution methods to determine the minimal inhibitory concentration (MIC) of antimicrobial substances. Nat. Protoc..

[26] O’Toole G.A. (2011). Microtiter Dish Biofilm Formation Assay. J. Vis. Exp..

[27] ASM MacConkey Agar Plates—Protocols. https://asm.org/protocols/macconkey-agar-plates-protocols.

[28] CLSI (2025). Performance Standards for Antimicrobial Susceptibility Testing (M100).

[29] Kim T.Y., Baek J.Y., Suh E., Lee J.-K., Yu H.-J., Ko J.-H., Huh H.J., Lee N.Y. (2025). Evaluation of the VITEK 2 AST-N439 card for susceptibility testing of novel β-lactam/β-lactamase inhibitor combinations and colistin in carbapenem-non-susceptible gram-negative bacilli. Microbiol. Spectr..

[30] Coudron P.E. (2005). Inhibitor-based methods for detection of plasmid-mediated AmpC β-lactamases in *Klebsiella* spp., *Escherichia coli*, and *Proteus mirabilis*. J. Clin. Microbiol..

[31] Magiorakos A.-P., Srinivasan A., Carey R.B., Carmeli Y., Falagas M., Giske C., Harbarth S., Hindler J., Kahlmeter G., Olsson-Liljequist B. (2012). Multidrug-resistant, extensively drug-resistant and pandrug-resistant bacteria: An international expert proposal for interim standard definitions for acquired resistance. Clin. Microbiol. Infect..

[32] Krumperman P.H. (1983). Multiple antibiotic resistance indexing of *Escherichia coli* to identify high-risk sources of fecal contamination of foods. Appl. Environ. Microbiol..

[33] WHO Antimicrobial Resistance. https://www.who.int/news-room/fact-sheets/detail/antimicrobial-resistance.

[34] Naghavi M., Vollset S.E., Ikuta K.S., Swetschinski L.R., Gray A.P., Wool E.E., Aguilar G.R., Mestrovic T., Smith G., Han C. (2024). Global burden of bacterial antimicrobial resistance 1990–2021: A systematic analysis with forecasts to 2050. Lancet.

[35] Sati H., Carrara E., Savoldi A., Hansen P., Garlasco J., Campagnaro E., Boccia S., Castillo-Polo J.A., Magrini E., Garcia-Vello P. (2025). The WHO Bacterial Priority Pathogens List 2024: A prioritisation study to guide research, development, and public health strategies against antimicrobial resistance. Lancet Infect. Dis..

[36] Calderaro A., Chezzi C. (2024). MALDI-TOF MS: A reliable tool in the real life of the clinical microbiology laboratory. Microorganisms.

[37] Elbehiry A., Aldubaib M., Abalkhail A., Marzouk E., Albeloushi A., Moussa I., Ibrahem M., Albazie H., Alqarni A., Anagreyyah S. (2022). How MALDI-TOF mass spectrometry technology contributes to microbial infection control in healthcare settings. Vaccines.

[38] Anorue M., Ejikeugwu C., Iroha C.S., David E.E., Nwabueze E.F., Iroha I.R. (2025). Extended spectrum beta-lactamase producing *Escherichia coli* encoding aminoglycoside and fluoroquinolone resistant genes in urinary tract infection patients in a tertiary hospital in Nigeria. BMC Infect. Dis..

[39] Lawal S.T., Usman F.A., Adams Z.A., Ogunbayo O.S., Ekwedigwe C.M., Jimoh R.O., Oladeru F.O., Osho O., Essiet U.U., Ajayi A. (2025). Genetic Determinants of Carbapenem and Fluoroquinolone Resistance in *Escherichia coli* Isolates of Clinical Origin. Infect. Chemother..

[40] Topa A.-E., Ionescu C., Pinzaru A., Mocanu E., Iancu A.M., Dumea E., Nitu B.F., Panculescu F.G., Cambrea S.C. (2025). Challenges in the Treatment of Urinary Tract Infections: Antibiotic Resistance Profiles of *Escherichia coli* Strains Isolated from Young and Elderly Patients in a Southeastern Romanian Hospital. Biomedicines.

[41] Alós J.-I., Serrano M.-G., Gómez-Garcés J.-L., Perianes J. (2005). Antibiotic resistance of *Escherichia coli* from community-acquired urinary tract infections in relation to demographic and clinical data. Clin. Microbiol. Infect..

[42] García-Meniño I., García V., Lumbreras-Iglesias P., Fernández J., Mora A. (2024). Fluoroquinolone resistance in complicated urinary tract infections: Association with the increased occurrence and diversity of *Escherichia coli* of clonal complex 131, together with ST1193. Front. Cell. Infect. Microbiol..

[43] Zlamal J.E., Leyn S.A., Iyer M., Elane M.L., Wong N.A., Wamsley J.W., Vercruysse M., Garcia-Alcalde F., Osterman A.L. (2021). Shared and unique evolutionary trajectories to ciprofloxacin resistance in gram-negative bacterial pathogens. mBio.

[44] de Lastours V., El Meouche I., Chau F., Beghain J., Chevret D., Aubert-Frambourg A., Clermont O., Royer G., Bouvet O., Denamur E. (2022). Evolution of fluoroquinolone-resistant *Escherichia coli* in the gut after ciprofloxacin treatment. Int. J. Med. Microbiol..

[45] Garoff L., Pietsch F., Huseby D.L., Lilja T., Brandis G., Hughes D. (2020). Population bottlenecks strongly influence the evolutionary trajectory to fluoroquinolone resistance in *Escherichia coli*. Mol. Biol. Evol..

[46] Toprak E. (2011). Evolutionary paths to strong antibiotic resistance under dynamically sustained drug stress. Nat. Genet..

[47] Huseby D.L., Pietsch F., Brandis G., Garoff L., Tegehall A., Hughes D. (2017). Mutation supply and relative fitness shape the genotypes of ciprofloxacin-resistant *Escherichia coli*. Mol. Biol. Evol..

[48] Jacoby G.A., Strahilevitz J., Hooper D.C. (2015). Plasmid-Mediated Quinolone Resistance. Plasmids: Biology and Impact in Biotechnology and Discovery.

[49] Blair J.M., Richmond G.E., Piddock L.J. (2014). Multidrug efflux pumps in Gram-negative bacteria and their role in antibiotic resistance. Future Microbiol..

[50] Webber M.A., Piddock L.J. (2003). The importance of efflux pumps in bacterial antibiotic resistance. J. Antimicrob. Chemother..

[51] Chetri S. (2023). The culmination of multidrug-resistant efflux pumps vs. meager antibiotic arsenal era: Urgent need for an improved new generation of EPIs. Front. Microbiol..

[52] Melnyk A.H., Wong A., Kassen R. (2015). The fitness costs of antibiotic resistance mutations. Evol. Appl..

[53] IDSA IDSA 2024 Guidance on the Treatment of Antimicrobial Resistant Gram-Negative Infections. https://www.idsociety.org/practice-guideline/amr-guidance/.

[54] Lázár V., Pal Singh G., Spohn R., Nagy I., Horváth B., Hrtyan M., Busa-Fekete R., Bogos B., Méhi O., Csörgő B. (2013). Bacterial evolution of antibiotic hypersensitivity. Mol. Syst. Biol..

[55] Imamovic L., Sommer M.O. (2013). Use of collateral sensitivity networks to design drug cycling protocols that avoid resistance development. Sci. Transl. Med..

[56] Podnecky N.L., Fredheim E.G., Kloos J., Sørum V., Primicerio R., Roberts A.P., Rozen D.E., Samuelsen Ø., Johnsen P.J. (2018). Conserved collateral antibiotic susceptibility networks in diverse clinical strains of *Escherichia coli*. Nat. Commun..

[57] Nichol D., Rutter J., Bryant C., Hujer A.M., Lek S., Adams M.D., Jeavons P., Anderson A.R., Bonomo R.A., Scott J.G. (2019). Antibiotic collateral sensitivity is contingent on the repeatability of evolution. Nat. Commun..

[58] Sørum V., Øynes E.L., Møller A.S., Harms K., Samuelsen Ø., Podnecky N.L., Johnsen P.J. (2022). Evolutionary instability of collateral susceptibility networks in ciprofloxacin-resistant clinical *Escherichia coli* strains. MBio.

[59] Liu D.Y., Phillips L., Wilson D.M., Fulton K.M., Twine S.M., Wong A., Linington R.G. (2023). Collateral sensitivity profiling in drug-resistant *Escherichia coli* identifies natural products suppressing cephalosporin resistance. Nat. Commun..

[60] Shehata A.A., Abd-Elfatah E.B., Elsheik H.E., Salman M.B., Khater A.S.I., El-Emam M.M.A. (2024). Epidemiological Features, Biochemical Indices, Antibiogram Susceptibility Profile and Biofilm Factor Genes of Klebsiella pneumoniae Isolated from Bovine Clinical Mastitis Cases. Pak. Vet. J..

[61] Aldred K.J., Kerns R.J., Osheroff N. (2014). Mechanism of quinolone action and resistance. Biochemistry.

[62] Chubiz L.M. (2023). The mar, sox, and rob systems. EcoSal Plus.

[63] Agnello M., Finkel S.E., Wong-Beringer A. (2016). Fitness cost of fluoroquinolone resistance in clinical isolates of *Pseudomonas aeruginosa* differs by type III secretion genotype. Front. Microbiol..

[64] Bhatnagar K., Wong A. (2019). The mutational landscape of quinolone resistance in *Escherichia coli*. PLoS ONE.

[65] Vanacker M., Lenuzza N., Rasigade J.-P. (2023). The fitness cost of horizontally transferred and mutational antimicrobial resistance in *Escherichia coli*. Front. Microbiol..

[66] FDA FDA Drug Safety Communication: FDA Updates Warnings for Oral and Injectable Fluoroquinolone Antibiotics Due to Disabling Side Effects. https://www.fda.gov/drugs/drug-safety-and-availability/fda-drug-safety-communication-fda-updates-warnings-oral-and-injectable-fluoroquinolone-antibiotics.

[67] WHO (2024). WHO Updates List of Drug-Resistant Bacteria Most Threatening to Human Health. https://www.who.int/news/item/17-05-2024-who-updates-list-of-drug-resistant-bacteria-most-threatening-to-human-health.

[68] Schuster S., Bohnert J.A., Vavra M., Rossen J.W., Kern W.V. (2019). Proof of an outer membrane target of the efflux inhibitor Phe-Arg-β-naphthylamide from random mutagenesis. Molecules.

